# Association of obesity risk SNPs in *PCSK1 *with insulin sensitivity and proinsulin conversion

**DOI:** 10.1186/1471-2350-11-86

**Published:** 2010-06-09

**Authors:** Martin Heni, Axel Haupt, Silke A Schäfer, Caroline Ketterer, Claus Thamer, Fausto Machicao, Norbert Stefan, Harald Staiger, Hans-Ulrich Häring, Andreas Fritsche

**Affiliations:** 1 Department of Internal Medicine, Division of Endocrinology, Diabetology, Angiology, Nephrology and Clinical Chemistry, Eberhard Karls University Tübingen, Member of the German Centre for Diabetes Research (DZD), Tübingen, Germany; 2 Department of Internal Medicine, Nutritional and Preventive Medicine, Eberhard-Karls-University Tübingen, Tübingen, Germany

## Abstract

**Background:**

Prohormone convertase 1 is involved in maturation of peptides. Rare mutations in gene *PCSK1*, encoding this enzyme, cause childhood obesity and abnormal glucose homeostasis with elevated proinsulin concentrations. Common single nucleotide polymorphisms (SNPs) within this gene, rs6232 and rs6235, are associated with obesity. We studied whether these SNPs influence the prediabetic traits insulin resistance, β-cell dysfunction, or glucose intolerance.

**Methods:**

We genotyped 1498 German subjects for SNPs rs6232 and rs6235 within *PCSK1*. The subjects were metabolically characterized by oral glucose tolerance test with glucose, insulin, proinsulin, and C-peptide measurements. A subgroup of 512 subjects underwent a hyperinsulinemic-euglycemic clamp.

**Results:**

The minor allele frequencies were 25.8% for SNP rs6235 and 6.0% for rs6232. After adjustment for sex and age, we found no association of SNPs rs6235 and rs6232 with BMI or other weight-related traits (all p ≥ 0.07). Both minor alleles, adjusted for sex, age, BMI and insulin sensitivity were associated with elevated AUC_proinsulin _and AUC_proinsulin_/AUC_insulin _(rs6235: p_additive model _≤ 0.009, effect sizes 8/8%, rs6232: p_dominant model _≤ 0.01, effect sizes 10/21%). Insulin secretion was not affected by the variants (different secretion parameters, all p ≥ 0.08). The minor allele of SNP rs6232 was additionally associated with 15% higher OGTT-derived and 19% higher clamp-derived insulin sensitivity (p_dom _≤ 0.0047), 4.5% lower HOMA_IR _(p_dom _= 0.02) and 3.5% lower 120-min glucose (p_dom _= 0.0003) independently of BMI and proinsulin conversion. SNP rs6235 was not associated with parameters of glucose metabolism.

**Conclusions:**

Like rare mutations in *PCSK1*, the more common variants tested determine glucose-stimulated proinsulin conversion, but not insulin secretion. In addition, rs6232, encoding the amino acid exchange N221D, influences insulin sensitivity and glucose homeostasis.

## Background

Proteolytic cleavage is an important step in the maturation of several hormones that are derived from inactive precursors [1,2]. One important enzyme that catalyzes this processing is prohormone convertase 1. This protease [3] is selectively expressed in neuronal and endocrine tissues [4] and is active within dense core secretory granules [2,3]. Among its numerous substrates [3,5] are key hormones and neuropeptides for the regulation of energy metabolism, including proopiomelanocortin, proglucagon, and proinsulin [1,4,6-9].

Patients with prohormone convertase 1 deficiency (MIM: #600955), a rare monogenic disease, suffer from childhood obesity, small intestinal absorptive dysfunction and various endocrine disturbances. They develop hypoadrenalism, hypogonadotrophic hypogonadism and abnormal glucose homeostasis with elevated proinsulin levels due to insufficient prohormone processing [9-12].

Genome-wide linkage studies identified a genomic locus on chromosome 5q to be associated with obesity [13-16]. Among other genes, this locus harbors *PCSK1*, and associations of two common single nucleotide polymorphisms (SNPs) within this gene (rs6232 and rs6235, minor allele frequency (MAF) = 5.4% and 27.3%, respectively) with obesity were reported [6].

Since prohormone convertase 1 is involved in proinsulin conversion and thereby in insulin maturation as well as in body weight regulation, the present study examined the association of the putatively obesity-related SNPs rs6232 and rs6235 in *PCSK1 *with the prediabetic traits insulin resistance, β-cell dysfunction, and glucose intolerance.

## Methods

### Participants

We studied 1498 non-diabetic subjects from southwestern Germany. The participants were selected from the ongoing Tübingen Family Study which currently includes ~2000 individuals with an increased risk for type 2 diabetes mellitus [17]. Selection was done based on the availability of all phenotype data used for the analyses. Especially proinsulin levels for all time points during the OGTT were not available for all subjects in the cohort. Most (68.5%) of these subjects had a family history of diabetes, i.e., at least one second-degree relative with type 2 diabetes. Informed written consent was obtained from all participants and the local Ethics Committee approved the protocol. The OGTT revealed that 73.0% of the non-diabetic participants had normal glucose tolerance, 10.0% had impaired fasting glycaemia, 9.6% had impaired glucose tolerance and 7.3% had impaired fasting glycaemia and impaired glucose tolerance.

### Genotyping

DNA from whole blood was isolated using a commercial DNA isolation kit (NucleoSpin, Macherey & Nagel, Düren, Germany). Genotyping was performed using the TaqMan assay (Applied Biosystems, Forster City, CA, USA). The TaqMan genotyping reaction was amplified on a GeneAmp PCR system 7000 and fluorescence was detected on an ABI PRISM 7000 sequence detector (Applied Biosystems). The genotyping success rates were 99.0% for rs6232 and 99.1% for rs6235. The genotypes were verified in 50 randomly selected subjects by bidirectional sequencing, and both methods resulted in 100% identical results. Both SNPs were in Hardy-Weinberg equilibrium (rs6232 p = 0.1 and rs6235 p = 0. 7). The SNPs were incompletely linked (D' = 0.887, r^2 ^= 0.145).

### OGTT

After an overnight fast, subjects ingested a solution containing 75 g glucose at 08:00 hours. Venous blood samples were obtained at 0, 30, 60, 90 and 120 min, and plasma glucose, insulin, C-peptide and proinsulin concentrations were determined.

### Hyperinsulinemic-euglycemic clamp

Clamp-derived insulin sensitivity was determined in a subgroup of 512 subjects by a hyperinsulinemic-eugylcemic clamp with measurement of glucose and insulin. The test was performed as described earlier [18].

### Analytical procedures

Plasma insulin and proinsulin were determined by commercial chemiluminescence assays for ADVIA Centaur (Siemens Medical Solutions, Fernwald, Germany). Blood glucose was measured using a bedside glucose analyzer (glucose oxidase method; Yellow Springs Instruments, Yellow Springs, OH, USA).

### Calculations

The area under the curve (AUC) AUC_Ins30_/AUC_Glc30 _was calculated as (Ins_0 _+ Ins_30_)/(Glc_0 _+ Glc_30_). First-phase insulin was determined from the OGTT as described earlier [19] by calculating 1283 + 1.829 × Ins_30 _- 138.7 × Glc_30 _+ 3.772 × Ins_0_. AUC of glucose, insulin, C-peptide and proinsulin during the OGTT were calculated according to the trapezoid method as: 0.5 × (c_0_/2 + c_30 _+ c_60 _+ c_90 _+ c_120_/2) with c = concentration. HOMA_IR _was calculated as (Glc_0 _× Ins_0 _× 2)/45. Insulin sensitivity during the OGTT was estimated as proposed by Matsuda and DeFronzo [20]: ISI = 10,000/√(Glc_0_·Ins_0_·Glc_mean_·Ins_mean_).

### Statistical analyses

Unless otherwise stated, data are given as arithmetic means ± SEM. Data were logarithmically transformed prior to statistical analysis. Hardy-Weinberg equilibrium was tested using χ^2 ^test. Differences in anthropometrics and metabolic parameters between genotypes were tested using multivariate linear regression analysis. *PCSK1 *SNP rs6235 and SNP rs6232 were examined under both an additive (add) and a dominant inheritance model (dom). However, only the dominant inheritance model was interpreted for SNP rs6232 because of this SNP's low MAF of 6% (only 5 participants were homozygous for the minor allele). All data were adjusted for gender and age. The glucose concentrations and insulin sensitivity indices were additionally adjusted for BMI, and proinsulin concentrations and insulin secretion parameters were additionally adjusted for OGTT-derived insulin sensitivity index and BMI.

Differences with a p-value < 0.05 were considered to indicate nominal associations. After correction for the two unlinked SNPs tested and the endpoints anthropometrics, insulin secretion, and insulin sensitivity (according to Bonferroni), results with values of p < 0.0085 were considered statistically significant. The JMP 7.0 (SAS Institute, Cary, NC, USA) statistical software package was used.

### Power calculation

Using two tailed t-test, the study was sufficiently powered (1-beta ≥ 0.8) to detect an effect size of 15% for SNP rs6235 and 22% for SNP rs6232 (dominant inheritance model, p = 0.05). Power calculations were performed using G*power 3.0 software available at http://www.psycho.uni-duesseldorf.de/aap/projects/gpower/.

## Results

The observed MAF for SNP rs6235 was 25.8% (reported 27.3% [6]) and for rs6232 6.0% (reported 5.4% [6]).

After appropriate adjustment, both minor alleles of the *PCSK1 *SNPs rs6232 and rs6235 were neither associated with BMI nor with weight-related traits, like waist circumference and total body fat (all p ≥ 0.07, tables 1 + 2). The frequency of obesity (BMI < 30 versus BMI ≥ 30 kg/m^2^) in our cohort was not influenced by the risk alleles (all p ≥ 0.4).

**Table 1:** Associations of *PCSK1 *SNP rs6232 with anthropometrics, metabolic parameters, and proinsulin conversion

SNP	rs6232 (encoding N221D)					
Genotype	AA	AG	GG	β_adj _± SEM	p_dom_	p_add_

N (f/m)	1305(875/430)	173(106/67)	5(3/2)	-	-	-

Age (years)	39 ± 0.4	40 ± 1	37.6 ± 4	-	-	-

BMI (kg/m^2^)	28.5 ± 0.2	28.3 ± 0.6	29.1 ± 6.1	-0.003 ± 0.001	0.8	1.0

Waist circumference (cm)	93.4 ± 0.5	93.1 ± 1.2	82.3 ± 3.5	-0.005 ± 0.007	0.6	0.9

Total body fat (%)	31.0 ± 0.3	29.6 ± 0.8	30.4 ± 7	-0.017 ± 0.013	0.07	0.4

Fasting glucose (mmol/l)	5.1 ± 0.1	5.1 ± 0.1	5.4 ± 0.4	-0.004 ± 0.004	0.3	0.2

Glucose_120-min _(mmol/l)	6.3 ± 0.1	5.9 ± 0.1	6.8 ± 1.2	-0.035 ± 0.010	**0.0003**	**0.0006**

Fasting insulin (pmol/l)	63 ± 1	56 ± 3	59.8 ± 33	-0.041 ± 0.019	0.0297	0.07

Insulin sensitivity, OGTT (AU)	16.3 ± 0.3	18.5 ± 0.9	25.8 ± 7	0.068 ± 0.020	**0.0010**	**0.0030**

Insulin sensitivity, clamp-derived (AU) (N = 512)	0.083 ± 0.003 (N = 443)	0.098 ± 0.007 (N = 68)	0.135 (N = 1)	0.085 ± 0.030	**0.0047**	-

HOMA_IR _(mU × mmol × l^-2^)	2.4 ± 0.1	2.1 ± 0.1	2.7 ± 1.8	-0.045 ± 0.020	0.0232	0.07

AUC_Ins30_/AUC_Glc30 _(×10^-9^)	41.2 ± 0.9	36.8 ± 2.0	28.1 ± 8.3	-0.001 ± 0.017	1.0	0.6

First-phase insulin (pmol/l)	1267 ± 24	1160 ± 53	1046 ± 273	0.015 ± 0.017	0.4	0.7

AUC_C-Pep_/AUC_Glc _(×10^-9^)	319 ± 3	319 ± 8	272 ± 29	0.020 ± 0.011	0.08	0.2

Proinsulin_0-min _(pmol/l)	5.8 ± 0.2	5.6 ± 0.4	12.8 ± 8.4	0.053 ± 0.038	0.2	0.2

Proinsulin_30-min _(pmol/l)	11.8 ± 0.4	11.9 ± 0.7	18.6 ± 3.7	0.073 ± 0.035	0.0377	0.0190

AUC_Proins _(pmol/l)	36.2 ± 1.0	37.2 ± 2.4	52.1 ± 12.7	0.074 ± 0.030	0.0141	**0.0089**

Fasting proinsulin/fasting insulin	0.128 ± 0.002	0.135 ± 0.003	0.179 ± 0.008	0.040 ± 0.039	0.3	0.3

Proinsulin_30-min_/insulin_30-min_	0.036 ± 0.004	0.035 ± 0.002	0.064 ± 0.004	0.079 ± 0.037	0.0346	0.0158

AUC_Proins_/AUC_Ins_	0.050 ± 0.016	0.058 ± 0.034	0.111 ± 0.017	0.079 ± 0.030	0.0094	**0.0026**

Data represent unadjusted means ± SEM. For statistical analysis, data were ln-transformed and adjusted for gender and age. Glucose concentrations and insulin sensitivity indices were additionally adjusted for BMI. Proinsulin values as well as insulin secretion parameters were additionally adjusted for BMI and insulin sensitivity (OGTT). AUC - area under the curve; β_adj _- effect sizes from the adjusted dominant model; BMI - body mass index; HOMA_IR _- homeostasis model assessment-estimated insulin resistance; OGTT - oral glucose tolerance test; p_add _- p-values from the additive inheritance model; p_dom _- p-values from the dominant inheritance model; SNP - single nucleotide polymorphism. p-values withstanding Bonferroni correction for multiple comparisons are given in bold.

**Table 2:** Associations of *PCSK1 *SNP rs6235 with anthropometrics, metabolic parameters, and proinsulin conversion

SNP	rs6235 (encoding S690T)					
Genotype	GG	GC	CC	β_adj _± SEM	p_dom_	p_add_

N (f/m)	817(537/280)	560(377/183)	108(72/36)	-	-	-

Age (years)	39 ± 1	40 ± 1	38 ± 1	-	-	-

BMI (kg/m^2^)	28.2 ± 0.3	28.7 ± 0.3	29.3 ± 0.8	0.016 ± 0.010	0.2	0.3

Waist circumference (cm)	92.9 ± 0.6	93.9 ± 0.7	94.9 ± 2.0	0.009 ± 0.007	0.2	0.9

Total body fat (%)	30.7 ± 0.4	31.0 ± 0.5	31.7 ± 1.0	0.014 ± 0.014	0.7	0.5

Fasting glucose (mmol/l)	5.1 ± 0.1	5.1 ± 0.1	5.1 ± 0.1	-0.004 ± 0.004	0.2	0.3

Glucose_120-min _(mmol/l)	6.2 ± 0.1	6.3 ± 0.1	6.3 ± 0.2	0.005 ± 0.011	0.7	0.8

Fasting insulin (pmol/l)	61 ± 2	64 ± 2	63 ± 5	0.021 ± 0.027	0.8	0.6

Insulin sensitivity, OGTT (AU)	16.8 ± 0.4	16.4 ± 0.5	16.0 ± 0.9	-0.018 ± 0.029	0.7	0.7

Insulin sensitivity, clamp-derived (AU) (N = 512)	0.088 ± 0.003 (N = 286)	0.080 ± 0.004 (N = 191)	0.091 ± 0.009 (N = 35)	-0.023 ± 0.021	0.3	0.4

HOMA_IR _(mU × mmol × l^-2^)	2.4 ± 0.1	2.5 ± 0.1	2.5 ± 0.2	0.017 ± 0.029	0.9	0.6

AUC_Ins30_/AUC_Glc30 _(×10^-9^)	40.4 ± 1.0	40.2 ± 1.3	46.0 ± 3.7	0.022 ± 0.026	0.9	0.6

First-phase insulin (pmol/l)	1247 ± 29	1251 ± 35	1383 ± 97	0.014 ± 0.024	1.0	1.0

AUC_C-Pep_/AUC_Glc _(×10^-9^)	318 ± 3.6	316 ± 4.4	343 ± 13	0.017 ± 0.014	1.0	0.2

Proinsulin_0-min _(pmol/l)	5.6 ± 0.2	5.9 ± 0.3	7.0 ± 0.9	0.081 ± 0.041	0.1	0.1

Proinsulin_30-min _(pmol/l)	11.2 ± 0.3	12.2 ± 0.7	16.5 ± 2.6	0.117 ± 0.038	0.0286	**0.0059**

AUC_Proins _(pmol/l)	34.2 ± 1.0	37.0 ± 1.5	49.1 ± 6.7	0.105 ± 0.034	0.0404	**0.0018**

Fasting proinsulin/fasting insulin	0.126 ± 0.004	0.129 ± 0.003	0.147 ± 0.014	0.062 ± 0.044	0.2	0.1

Proinsulin_30-min_/insulin_30-min_	0.036 ± 0.005	0.035 ± 0.002	0.047 ± 0.011	0.105 ± 0.041	0.0208	0.0123

AUC_Proins_/AUC_Ins_	0.048 ± 0.001	0.053 ± 0.003	0.068 ± 0.011	0.085 ± 0.035	0.0324	0.0090

Data represent unadjusted means ± SEM. For statistical analysis, data were ln-transformed and adjusted for gender and age. Glucose concentrations and insulin sensitivity indices were additionally adjusted for BMI. Proinsulin values as well as insulin secretion parameters were additionally adjusted for BMI and insulin sensitivity (OGTT). AUC - area under the curve; β_adj _- effect sizes from the adjusted dominant model; BMI - body mass index; HOMA_IR _- homeostasis model assessment-estimated insulin resistance; OGTT - oral glucose tolerance test; p_add _- p-values from the additive inheritance model; p_dom _- p-values from the dominant inheritance model; SNP - single nucleotide polymorphism. p-values withstanding Bonferroni correction for multiple comparisons are given in bold.

**Figure 1:**
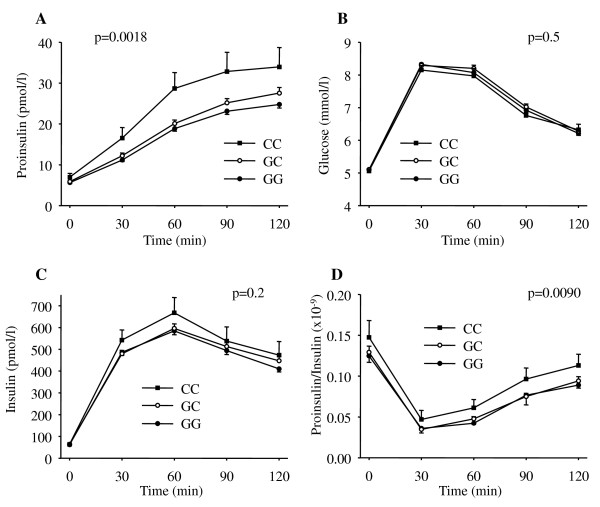
**Plasma proinsulin (A), glucose (B), and insulin (C) concentrations as well as proinsulin-to-insulin ratio (D) during OGTT in carriers of the *PCSK1 *SNP rs6235**. Data are given as means +/- SEM. P-values for AUC adjusted for gender, age, BMI, and insulin sensitivity are given. Black boxes: homozygous carriers of the minor allele; white circles: heterozygous carriers; black circles: homozygous carriers of the major allele.

None of the SNPs revealed association with fasting proinsulin concentrations (all p ≥ 0.1, tables 1 + 2). However, upon glucose ingestion, subjects carrying the minor alleles showed an association with higher proinsulin concentrations (rs6232 nominally associated, p_dom _= 0.0104; rs6235 significantly associated with AUC_Proins_, p_add _= 0.0018; adjusted effect sizes 10/8%; tables 1 + 2) and nominally lower proinsulin-to-insulin conversion (AUC_Proins_/AUC_ins_) compared to those with the major allele (rs6232, p_dom _= 0.0094; rs6235, p_add _= 0.0090; adjusted effect sizes 21/8%; tables 1 + 2, figure 1A+1D) and this finding was consistent when adjusting for insulin sensitivity or not. Both variants were not associated with differences in insulin secretion (all indices used p ≥ 0.08, tables 1 + 2), and SNP rs6235 was furthermore neither associated with glucose nor with insulin levels during the OGTT (all p ≥ 0.2, figure 1B and 1C).

**Figure 2:**
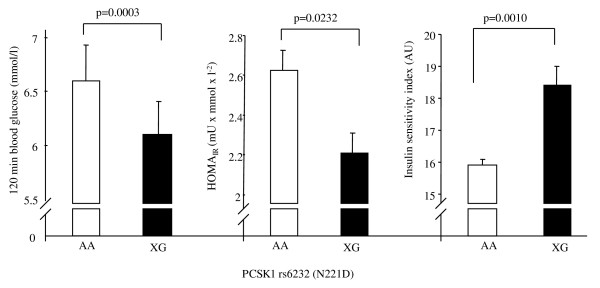
**Association of rs6232 (N221D) in *PCSK1 *gene with insulin sensitivity derived from OGTT, insulin resistance derived from the fasting state (HOMA_IR_) and 120-min glucose**. Data were adjusted for BMI, age and gender (dominant inheritance model).

In addition to these findings, the minor allele of SNP rs6232, but not that of SNP rs6235, was significantly associated with decreased 120-min blood glucose (p_dom _= 0.0003, table 1, figure 2) and nominally associated with decreased fasting insulin levels (p_dom _= 0.0297, table 1). Insulin sensitivity derived from the OGTT was significantly increased (p_dom _= 0.0010, adjusted effect size 15%, table 1, figure 2) and insulin resistance derived from fasting state (HOMA_IR_) was accordingly nominally decreased in carriers of the minor allele (p_dom _= 0.0232, table 1 and figure 2). The effect on OGTT-derived insulin sensitivity remained significant even after adjustment for gender, age, BMI, AUC_Proins_/AUC_Ins_, and first-phase insulin secretion (p_dom _= 0.0033).

To confirm that the association with insulin sensitivity did not solely reflect altered insulin levels due to impaired prohormone processing, we analyzed insulin sensitivity measured by exogenous administration of insulin during hyperinsulinemic-euglycemic clamp. Again, SNP rs6232, but not SNP rs6235 was associated with significantly increased insulin sensitivity (p_dom _= 0.0047; adjusted effect size 19%; table 1).

To analyze subjects with low risk of type 2 diabetes mellitus versus those with a high risk, we stratified for family history of diabetes. This stratification did not produce consistent results, probably due to the limited power of the subgroups.

## Discussion

### Association of PCSK1 with weight-related traits

In our cohort, the reported association of the common variants rs6232 and rs6235 within the *PCSK1 *gene with BMI and obesity could not be replicated. This is most likely explained by the limited power of our study to detect relatively small effect sizes of the SNPs on BMI and obesity. The effect sizes detectable in our study were as small as 15%, and for the established obesity risk locus *FTO *an effect size of about 10% could be demonstrated [21].

### Association of PCSK1 with proinsulin levels

In the present study, we found that the minor alleles of both candidate SNPs in the *PCSK1 *gene are associated with significantly higher glucose-stimulated proinsulin levels indicating reduced proinsulin conversion, while insulin secretion was unaffected. These findings were not altered by adjustment for insulin sensitivity.

Basal proinsulin levels were unaffected by the variants in *PCSK1*. Thus, under basal conditions the enzyme cleaves sufficient amounts of proinsulin. However, in the state of much higher secretory demands following a glucose load, proinsulin levels and proinsulin-to-insulin ratios were increased in carriers of both SNPs and this points to insufficient insulin maturation.

Both SNPs in the *PCSK1 *gene alter the amino acid sequence of the protein. SNP rs6232 changes asparagine to aspartic acid (N221D) in the catalytic domain and leads to a 10% reduction of activity, while SNP rs6235 substitutes serine by threonine (S690T) without any effect on the enzymatic activity [6]. Reduced enzymatic activity due to SNP rs6232 can plausibly explain this observation, though the mechanism is less clear in the case of the other SNP, rs6235. The substituted amino acid by that variation could possibly influence binding of regulators of the enzyme's activity, its localization within the β-cell, or the available number of prohormone convertase 1 molecules via altered stability. All these effects could theoretically influence prohormone conversion.

Although both *PCSK1 *SNPs affect proinsulin levels, they do not alter insulin secretion after glucose stimulation. Thus, the enzyme does not represent the rate-limiting step in insulin secretion which is in contrast to SNPs in diabetes risk genes which influence both proinsulin conversion and insulin secretion [22].

### Association of PCSK1 SNP rs6232 with insulin sensitivity

Furthermore, the putative obesity risk allele of SNP rs6232 was unexpectedly associated with increased insulin sensitivity (fasting, OGTT-derived, and hyperinsulinemic euglycemic-clamp derived) and reduced 120-min glucose levels independently of BMI and proinsulin conversion. The similar findings with different independent measurements of insulin sensitivity as well as the inverse influence on 120-min blood glucose argue against a mere statistical type 1 error.

Since only SNP rs6232 in the *PCSK1 *gene, the one that decreases the enzyme's catalytic activity [6], influences insulin sensitivity, independently of BMI and proinsulin conversion, and since there is a long list of potential substrates of the prohormone convertases [3-5], it seems reasonable to speculate that the enzyme also cleaves factors that modulate insulin sensitivity (e.g. BNDF [23]).

In most cases, obesity is associated with insulin resistance. However, a proportion of overweight/obese persons display a phenotype of obesity with is metabolically benign and characterized by high insulin sensitivity [17]. Since the minor allele of SNP rs6232 was associated with higher insulin sensitivity in our study and with obesity in a large metaanalysis including more then 13,000 subjects [6], we hypothesize that this allele may promote a kind of obesity, which is more benign.

Increased proinsulin levels are reported to be a marker of insulin resistance [24]. Interestingly, for carriers of *PCSK1 *SNP rs6232 it is the other way round: Those with higher proinsulin concentrations during the OGTT have also increased insulin sensitivity. Similar findings are reported in another publication where insulin resistance was associated with enhanced proinsulin processing in subjects with normal glucose tolerance [25]. Hence, increased proinsulin concentration can not generally be seen as a marker of insulin resistance.

### Limitations

Our study was underpowered to detect the previous reported associations of the *PCSK1 *variants with obesity. The associations with proinsulin conversion and insulin sensitivity found in this study clearly need replication in other cohorts, in particular, the unexpected association of SNP rs6232 with insulin sensitivity. Furthermore, efforts should also be undertaken to identify prohormone convertase 1 substrates that alter insulin sensitivity and to further characterize these mechanisms. Studies on the molecular level could additionally help to answer the question how *PCSK1*'s SNP rs6235 influences proinsulin levels without changing the enzyme's activity.

## Conclusions

In conclusion, our data suggest that the common genetic variants rs6232 and rs6235 within *PCSK1 *determine glucose-stimulated proinsulin conversion, but not insulin secretion. In addition, rs6232, encoding the mutation N221D, influences glucose homeostasis and insulin sensitivity independently of BMI and proinsulin concentrations.

## Abbreviations

HOMA_IR_: homeostasis model assessment-estimated insulin resistance; MAF: minor allele frequency; p_add_: p-values from the additive inheritance model; *PCSK1*: prohormone convertase 1 gene; p_dom_: p-values from the additive inheritance model; SNP: single nucleotide polymorphism

## Competing interests

The authors declare that they have no competing interests.

## Authors' contributions

MH and AH performed the analyses and wrote the manuscript. MH, AH, CT, and CK performed the clinical investigations. FM was responsible for genotyping. NS, HS, and AF controlled the analyses and discussed the paper. NS, H-UH, and AF directed the study. All authors read and approved the final manuscript

## Pre-publication history

The pre-publication history for this paper can be accessed here:

http://www.biomedcentral.com/1471-2350/11/86/prepub
